# Antecedents and Consequence of the Use of Channel Power: Evidence From China Petrochemical Industry

**DOI:** 10.3389/fpsyg.2022.927293

**Published:** 2022-06-13

**Authors:** Jing Tian, Qianmin Sun, Xiaoyu Deng

**Affiliations:** ^1^Head Office, Agricultural Bank of China, Beijing, China; ^2^Department of Marketing, Business School, Beijing International Studies University, Beijing, China; ^3^Department of Marketing, Business School, Beijing Technology and Business University, Beijing, China

**Keywords:** petrochemical industry, channel dependence, dependence symmetry, channel power, channel satisfaction

## Abstract

Inconsistency exists in extant research on the relation of channel dependence, the relation of channel power, and channel satisfaction. This study, taking Sinopec as the research object, explores the relation between channel dependence, the use of channel power, and channel satisfaction. The results reveal that dependence symmetry plays a moderating role in the relation between channel dependence and the use of coercive power. Specifically, with the increase of dependence symmetry, the influence of channel dependence on the use of coercive power becomes weaker. The authors also find that the use of coercive power is negatively related with the dealer’s channel satisfaction. However, the relation strength is weaker than related studies in Western cultural context.

## Introduction

In the studies of channel behavior, channel power has always been a focus for many scholars. Since the 1970s, western scholars have conducted in-depth research on channel power, the source of channel power, and the relationship between power and conflict, with an abundance of theoretical results (Gaski, 1984; Frazier, 1999; Hopkinson and Blois, 2014). Western scholars generally believe that channel power refers to the power of a channel member in controlling strategic marketing decision variables of channel members at a different level in a given channel system (El-Ansary and Stern, 1972; Hunt and Nevin, 1974; Frazier and Summers, 1986; Zhuang and Zhou, 2004). A concept corresponding to channel power is the use of channel power, which refers to the specific use of various powers owned by channel members (Frazier and Summers, 1984; Yi and Li-ping, 2006). Obviously, channel power and the use of channel power are not the same concepts. Channel members with channel power do not necessarily use it, but the use of channel power must be based on the ownership of channel power. Compared with channel power, the use of channel power receives much less attention, and there are no consistent conclusions on many research topics.

First, what is the basis for the channel members to use their power? According to the dependency-power theory, channel power depends on how dependent a channel member is on another. In other words, A has power over B because B depends on A (Emerson, 1962; Wolk and Skiera, 2009). Subsequent studies basically confirmed the positive relation between channel power and channel dependence (Etgar, 1976; Lusch and Brown, 1982; Skinner and Guiltinan, 1985; Heide and John, 1988). However, there is wide disagreement on the relationship between channel dependence, power, and the use of channel power. For instance, the empirical study by Frazier and Summers (1986) shows that the more power manufacturers have, the more likely they will use non-coercive influential tactics (demand and information exchange), and the less likely they will use coercive influential tactics (threat, legal defense, and promise). Similar conclusions were drawn by Frazier and Rody (1991); Boyle and Dwyer (1995), Runyan et al. (2010), and Zhuang and Zhou (2002). Meanwhile, Frazier et al. (1989) found that in a seller’s market, the dealer’s dependence on a manufacturer is positively correlated with the manufacturer’s use of coercive power. Similar conclusions were drawn by Kale (1986); Anderson and Narus (1990), and Gassenheimer and Ramsey (1994).

Second, what are the consequences of channel members’ use of power? In general, the quality of channel relationship is the outcome variable of evaluating the use of channel power, such as channel satisfaction, trust, commitment, and long-term willingness to cooperate (Morgan and Hunt, 1994; Yang et al., 2012). There are also differences in conclusions from related researches. For example, most existing studies argue that the use of coercive power is negatively correlated with channel satisfaction, while the use of non-coercive power is positively correlated with channel satisfaction (Hunt and Nevin, 1974; Lusch, 1976; Dwyer, 1980; Gaski and Nevin, 1985).

However, a cross-cultural study focused on Japan and the United States conducted by Johnson et al. (1993) shows that United States suppliers’ use of coercive power on Japanese dealers is positively correlated with Japanese dealers’ satisfaction perception. Slater and Robson (2012) also found that Japanese–Western alliances have a special attribution in many respects in the marketing channel due to the Japanese unique culture. In addition, Zhang and Marsha’s (2004) study of dealers in the apparel industry in China also found that the use of punitive power has a positive effect on channel members’ satisfaction. Su et al. (2009) found that task environment, social relations, and institutional norms can influence channel communication in China. Therefore, it is necessary to conduct more empirical studies on the use of channel power and channel relationship in different cultures (Samaha et al., 2014). In our study, we use the data from China, which has a unique cultural characteristic. Hofstede (2001) came up with the conclusion that countries with high uncertainty aversion tend to be more collectivist and avoid conflicts, based on his study on micro-perspective of customer psychology by surveying IBM employees from over 30 countries, and the survey targeted for China residents shows that Chinese residents are in the extreme value of long-term orientation with fairly high collectivism, presenting the cultural characteristics formed under the influence of long-term institutional and cultural instability. The following literature further supports this result by extending the survey or exploring new attributions to the present studies (Schwartz, 2006; Trompenaars and Hampden-Turner, 2011). Thus, in this study, we would consider the channel power under Chinese culture.

In this study, we explore the petrochemical industry, a unique industry in China. The petrochemical industry in China is oligarch and there are mainly two corroborations: China Petroleum & Chemical Corporation and Chinese National Petroleum Corporation. In our study, we use the survey from China Petroleum & Chemical Corporation. There are two kinds of channel distributions of the corporation: self-operated gas stations and franchises, and we focus on the latter one. In China, also in many emerging markets, the petrochemical industry is a massive management and highly competitive, and the channel relation between supplier and dealer is more complicated in this oligarch industry (Nolan and Zhang, 2002). Therefore, it is important to study the channel relation in the industry, including channel power, channel power behavior, and channel satisfaction.

In the study, we mainly explore two important parts: firstly, it argues that an important reason for the ambiguous relationship between channel dependence and the use of channel power is that existing studies on channel dependence often only consider a single dimension: one channel member’s dependence on another. However, it is a simple logic that A’s dependence on B is not necessarily related to B’s independence on A. This study takes dependence symmetry into account when evaluating channel dependence structure, and finds that channel dependence symmetry acts as an important moderator in the relationship between channel dependence and the use of power. Second, the use of channel power will influence channel performance. Secondly, in particular, in the context of Chinese culture, its effect on channel satisfaction somewhat contradicts existing studies. This study will provide more empirical support for relevant theoretical research, especially in the petrochemical industry. This study might contribute to the theoretical result by exploring the antecedents, which are dependence and consequence, which is the satisfaction, of channel power. Besides, we would also consider the moderator, the dependence symmetry, into the analysis. More importantly, we emphasize the concept of channel dependence symmetry and give a possible explanation for inconsistency in existing studies on channel dependence and the use of power. Moreover, our research would also contribute to the industry that it could provide useful information to the managers about how to deal with channel communication in many respects. Particularly, our research makes managerial implications to the suppliers and dealers within the petrochemical industry by providing specific suggestions in marketing channel management.

## Theoretical Background and Research Hypothesis

According to the SCP paradigm of the Harvard school, the market structure determines corporate behavior in the market, which determines the economic performance in all aspects of market operation (Bain, 1951, 1956). This study believes that the use of channel power originates from channel dependence structure, and the use of channel power will affect the quality of channel relationship. This study mainly measures the quality of channel relationship through channel satisfaction.

### Channel Dependence and the Use of Channel Power

Emerson’s (1962) study of power-dependence from the perspective of sociology holds that power is equal to and derived from dependence, which means the A’s power to B depends on B’s dependence on A. Meanwhile, Emerson explored the origin of the dependence, arguing that B’s dependence on A was positively correlated with B’s motivational investment for A’s adjusted goal, and negatively correlated with B’s likelihood of achieving its goal outside the A–B relationship. Based on Emerson’s (1962) study, many marketing scholars introduced channel dependence into their studies of channel power and argued that power can be deemed as the degree of dependence of one channel member on another (El-Ansary and Robicheaux, 1974; Frazier, 1983; Stern and El-Ansary, 1992; Wolk and Skiera, 2009; Jain et al., 2014; Zhang et al., 2016). In marketing channel systems, channel members often need to rely on other members to achieve their common goal because the expertise of each channel member varies (Stern and El-Ansary, 1992). Frazier (1983) gives a relatively formal definition of channel dependence: channel dependence refers to the extent to which channel members need to maintain a cooperative relationship with other related members to achieve their desired goals in a channel system. In studies of channel dependence and power, quite a few empirical studies proved the positive relationship between power and dependence. For instance, Etgar (1976) examined the relationship between channel leader’s power, power base, dependence, and offsetting power. It was found that the channel leader’s power is positively correlated with his power base and dependence, and negatively correlated with channel members’ offsetting power. While proving the positive effect of dependence on power, Skinner and Guiltinan (1985, 1986) also found that the increase in channel members’ external connections will reduce the level of dependence in the original channel relationship, thereby indirectly reducing the power level of trading partners in the original channel relationship. Nyaga et al. (2010) explore the dependence of supplier and dealer and found that the dependence has a positive effect on channel power for both of them. Jain et al. (2014) explored that trust plays an important role in the relation dependence and power, and it can moderate the impact in some conditions. Johnston et al. (2018) pointed out that the influence strategies show stronger positive impacts of non-coercive result from a higher appreciation for supportive attempts, whereas the higher negative sensitivity to coercive can be attributed to the low acceptability of forceful influences.

Frazier and Summers (1984) distinguished between the possession of channel power and the use of it. They believed that the use of channel power is a communication strategy adopted by channel members to change the behavior of other channel members. It is a specific use of the various powers they possess. Frazier and Summers (1984) further generalized six behavioral influence strategies, including information exchange, suggestion, commitment, threat, legal means, and request. Depending on whether the source company achieves its ultimate goal by changing the target company’s recognition of its expected behavior, the six influence strategies can be divided into coercive influence strategy and non-coercive influence strategy (Kale, 1986; Yi and Li-ping, 2006). The former strategy pertains to the use of commitments, threats, and legal means, while the latter pertains to the use of requests, information exchange, and advice strategies. In general, if the target channel member does not comply with coercive influence strategies, it usually leads to the user of channel power imposing negative sanctions on the target channel member (Frazier and Summers, 1986). Scheer et al. (2015) indicated that the non-coercive behavior can do a better job in channel communication at the beginning of the conflicts and can help to maintain a long-term friendly relationship between channel members. Sharma et al. (2022) also found that the supply chains’ structure significantly influences players’ sustainability efforts and profits in a supply chain, and the channel power would be affected by supply chain agents’ sustainability efforts and pricing strategies.

In the context of China, we believe that channel dependence is usually positively correlated with the use of non-coercive power. Firstly, channel members essentially share a strong common interest. They prosper and decline together. When trying to show its influence, a channel member is more willing to use non-coercive powers such as experts, information, and related influences, and this willingness is strengthened by China’s unique cultural values. It is generally believed that China’s culture is a collectivist one and holds a high degree of uncertainty avoidance (Hofstede, 1983; Schwartz, 2006; Trompenaars and Hampden-Turner, 2011), which prompted channel members to focus more on the predictability and stability of channel relationships (Kale and McIntyre, 1991; Hofstede, 2001; Samaha et al., 2014). Sternquist et al. (2002) even compared the relationship between channel members to the one between families. Channel members support each other to form a relatively stable network of relationships. Therefore, against the backdrop of the norms of such as a cultural system, channel members prefer to influence others through “soft power” to maintain a stable “relationship.” In particular, when the buyer is highly dependent and holds a weak position in the market, the party relied on finds more willingness and responsibility to support “one of its own” in its relationship network through the use of non-coercive power (Zhuang and Zhou, 2004). Su et al. (2009) highlight that in China, the coercive power behavior would not be easily used except for some extremely serious conditions because coercive behavior is considered hostile in Chinese culture. Moreover, Jia and Wang (2013) reviewed and integrated studies of marketing channels in the Chinese context from an institution-based perspective and explored the impact of Chinese institutional environments on marketing channels.

Secondly, highly dependent dealers usually indicate that suppliers have more resources, including soft powers such as knowledge and brands, and thus the party relied on is also more capable of using non-coercive power. In summary, when channel dependence is quite heavy, suppliers in the channel members have a greater willingness and ability to use non-coercive power. Such an argument is supported by many empirical studies. For instance, Gaski and Nevin (1985) found that when a channel member enjoys a high level of power, the often-used power is non-coercive power rather than coercive power. Frazier and Summers (1986) found that manufacturers’ power is positively correlated to their use of non-coercive influence strategies (requirements and information exchange). In the context of China, Zhuang and Zhou (2002) found that in the relationship between a department store in a city and its suppliers, the greater power one has, the more likely it is to use non-coercive power. Zhang et al. (2016) figure out that in a dynamic market, the channel power of the dealer and supplier should use a gentle way to solve problems if possible. Xuan et al. (2020) found that similarities in suppliers’ and buyers’ distributive fairness perception have consequential effects on suppliers’ non-coercive power use and buyers’ attitudinal and behavioral responses.

Therefore, this paper proposes the following assumption.

H1: *In China’s corporate channel relationship, dealer’s channel dependence is positively correlated to supplier’s use of non-coercive channel power.*

In the context of China, this study believes that channel dependence and the use of coercive power are negatively correlated. First, the use of coercive power is often viewed as an unfriendly behavioral strategy that tends to undermine the trust and commitment among channel members, even causing channel conflict (Kumar, 2005; Runyan et al., 2010), thus it is not recommended. Particularly under the influence of traditional Chinese culture, the harmonious interpersonal relationship has become an important goal of social development (Zhuang and Xi, 2003). In business environment, “harmony brings money,” as a Chinese idiom goes, while the use of coercive channel power tends to “spoil harmony.” In particular, when the dealer has greater channel dependence and the supplier has great market strength, the strong bullying the weak is even less likely to be endorsed. Second, stronger channel dependence of the dealer indicates that the party relied upon has enough resources that the dealer does not possess, and it is more than capable of punishing and deterring the dealer, making it less likely that the dealer would stop cooperating with them. This also makes it less necessary for the party relied upon to use coercive power. Due to these two factors, channel members relied on can achieve their goal of channel communication and management without using coercive power. This conclusion is also supported by many empirical studies. For instance, Frazier and Summers (1986) found that manufacturers’ power is negatively related to their use of coercive influence strategies (threats, legal defenses, and promises). Research by Frazier and Rody (1991) found that members who enjoy a power advantage tend to use influence strategies like information exchange and advice more than coercive strategies like commitments, threats, and legal means. Boyle and Dwyer’s (1995) study of suppliers and dealers of industrial goods also found that the greater power a supplier has, the more likely it is to use information exchange rather than strategies like legal defense and threats. Su et al. (2009) found that in the eastern countries, the dealer would choose not to use coercive power to show the friendly attitude to the supplier and enhance its competitive advantage. Leonidou et al. (2017) suggest that the coercive power sometimes could diminish the relationship between channel members. Jia et al. (2021) suggested that procedural fairness perception strengthens the effect of non-coercive influence on opportunism tendency, and distributive fairness worsens the harmful effect of coercive influence on the reseller’s opportunism tendency. Based on this, this study proposes the following assumption.

H2: *In China’s corporate channel relationship, dealer’s channel dependence is negatively correlated to supplier’s use of coercive channel power.*

### Moderation by Channel Dependence Symmetry

Nevertheless, many other empirical studies drew opposite conclusions to H1 and H2. Frazier et al. (1989) found in their study of behavior in marketing channels for industrial goods in developing countries that dealers’ dependence on manufacturers is positively correlated to manufacturers’ use of coercive power. Kale (1986) also found that in a seller’s market, manufacturers’ power is positively correlated to its use of coercive influence strategies. Gassenheimer and Ramsey’s (1994) study of the channel relationship formed by three suppliers and one dealer found that the greater dealer’s relative dependence is, the more coercive power suppliers use, and such coercive power is mostly used by the most important suppliers. This study believes that the differences in existing research findings are related to the researchers’ construction of channel dependence. Existing research often only considers one aspect of dependence, i.e., A’s dependence on B, while B’s dependence on A draws little attention. After the 1990s, the concept of dependence has been upgraded to a bilateral level (Lawler and Yoon, 1996). In addition to considering A’s dependence on B, this study also considers the symmetry of A and B’s dependence on each other and believes that dependence symmetry plays a moderating role in the relation between dependence and the use of power. A’s dependence on B indicates that A can obtain the expected utility from B, and such an expectation originates from the fact that B possesses very valuable resources for A to achieve its goal. In particular, when a dealer’s channel dependence negatively affects the supplier’s coercive power, and if there’s great channel dependence for the dealer and great channel dependence symmetry for both sides, i.e., great channel dependence for the supplier, then the supplier and the dealer each possess very valuable resources for each other to achieve their objectives, and neither holds an obvious market advantage. Since the demand for cooperation is strong between the two, “neither can thrive without the other,” and they share “an equal footing” in the market, both sides will add necessary measures like legal actions, contracts, promises, and even defenses, so as to influence the other’s market behavior (Zhuang and Xi, 2003). It can also claim that when the symmetry increases, even the dealer uses the coercive power, the strength, and the threatening power is less presented especially in the eastern culture (Su et al., 2009). Johnston et al. (2018) found the degrees of appreciation and acceptability by the perceived significance of benefits or damages, expectations, and tolerance levels for channel strategies. Kumar and Venkatesan (2021) emphasize the moderator of brand recognition on channel power and explored the consumer factor behind the trends in the retail industry.

Therefore, this study proposes the following assumptions:

H3: *In China’s corporate channel relationship, channel dependence symmetry moderates the influence of dealer’s channel dependence on the supplier’s use of coercive power. As channel symmetry increases, the dealer’s channel dependence will have less effect on the supplier’s use of coercive power.*

Meanwhile, when there’s great channel dependence for the dealer and great dependence symmetry for both sides, the suppliers often only have some advantages in basic non-coercive power resources such as information, technology, brand, and reputation, while other advantages are often owned by the dealer (Zhuang and Xi, 2003; Nyaga et al., 2010; Watson et al., 2015). Zhang et al. (2019) found that the manufacturer would give up the pricing power with the change of the exogenous wholesale price, and such willingness are different with the manufacturer’s information accuracy under one-sided information-sharing mechanism and two-sided information-sharing mode. For example, a gas supplier manufacturer may have an advantage in its brand and technology, but the gas station chain in the distribution channel often has obvious advantages in information, distribution networks, etc., resulting in a dependence structure where there is a great channel dependence for the dealer and great dependence symmetry for both sides. In this case, suppliers often can only mobilize some basic non-coercive power resources and use limited non-coercive power.

Therefore, this study proposes:

H4: *In China’s corporate channel relationship, channel dependence symmetry moderates the influence of dealer’s channel dependence on supplier’s use of non-coercive power. As channel symmetry increases, dealer’s channel dependence will have less effect on the supplier’s use of non-coercive power.*

### Use of Channel Power and Channel Satisfaction

The importance of the use of channel power lies in its influence on the quality of channel relationship and channel performance. For example, Lusch’s (1976) study of car manufacturers and dealers in the United States market found that the use of channel power affects channel conflicts. The use of rewarding and punitive power often causes conflicts, while the use of experts and related powers reduces conflict.

Channel satisfaction is an important variable for evaluating the quality of channel relationship. For the relationship between the use of channel power and channel satisfaction, a common research finding is that the use of non-coercive power is positively correlated with channel satisfaction (Hunt and Nevin, 1974; Lusch, 1976; Dwyer, 1980; Gaski and Nevin, 1985). However, there is wide disagreement on the relationship between coercive power and channel satisfaction. Most studies argued that the use of coercive power will lead to channel conflicts and thus reduce channel satisfaction (e.g., Hunt and Nevin, 1974; Lusch, 1976; Frazier and Summers, 1986; Griffith and Zhao, 2015; Krafft et al., 2015). Skarmeas et al. (2016) found that the coercive power would lead to a negative outcome between channel members, especially when the conflicts already existed. Meanwhile, some empirical studies yielded opposite results. For instance, a cross-cultural study on Japan and the United States conducted by Johnson et al. (1993) suggested that United States suppliers’ use of coercive power on Japanese dealers is positively correlated with the Japanese dealers’ perceived satisfaction. Sharma et al. (2022) also found that the supply chains structure significantly influences players’ sustainability efforts and profits in a supply chain, and the channel power would be affected by supply chain agents’ sustainability efforts and pricing strategies.

Moreover, Zhang and Marsha’s (2004) study of Chinese dealers found that the use of punitive power is positively correlated with channel members’ satisfaction. However, the conclusion of those studies is mostly an “accident,” for which the authors did not give a convincing explanation, except for indicating that cultural factors may be in play. Meanwhile, Lee’s (2001) research in Chinese cultural background again supports the conclusion that the use of coercive power would reduce channel satisfaction. This study argues that the use of coercive channel power is an external coercive behavior by the party relied on. The relying party has no choice but to submit, but psychologically it’s unwilling to do so, thus satisfaction would diminish. Non-coercive channel power is that the relying party accepts the proposal of the party relied on from the heart. Such a proposal would be well accepted and carried out by the relying party, thus satisfaction will increase. Such an argument is convincing both from a logical standpoint and from most empirical studies. Su et al. (2009) also found similar results that coercive power does not fit Chinese culture in many situations. Liu et al. (2021) suggested that the supply chain decision-making, which is an important issue of channel management, is related to consumer’s preference. Wang et al. (2021) figured out that the retailer’s sharing information behavior always benefits for the collector and the entire supply chain, which contributes to the channel satisfaction in an average level.

Therefore, this study proposes the following assumptions:

H5: *In China’s corporate channel relationship, supplier’s use of coercive channel power diminishes the dealer’s satisfaction.*

H6: *In China’s corporate channel relationship, the supplier’s use of non-coercive channel power increases the dealer’s satisfaction.*

**FIGURE 1:**
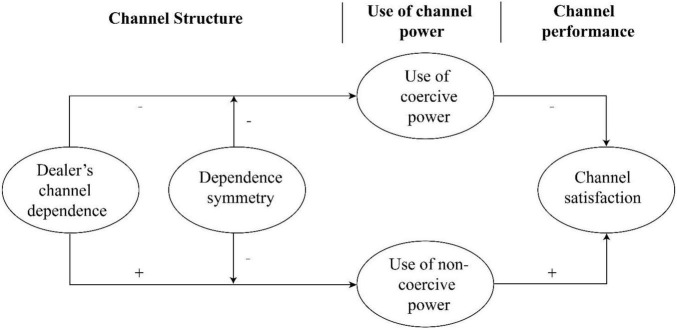
Research framework.

The research framework of this study is shown in Figure 1.

## Research Design

### Samples

The petrochemical industry is an oligarch industry in China. Within the industry, there are two main companies: China Petroleum & Chemical Corporation and Chinese National Petroleum Corporation. Though the industry is oligarch, the companies in this industry face high competition and complicate the relationship between suppliers and dealers, which leads to great management investment. In this study, we choose the China Petroleum & Chemical Corporation to collect data. The company has two-channel distribution modes: self-operated gas stations and franchises. In this study, we mainly focus on the franchises. In this study, the relationship between China Petroleum & Chemical Corporation (hereinafter referred to as “Sinopec”) and non-self-operated gas stations (jointly operated gas stations, chartered gas stations, social gas stations) is selected as the research object, and gas stations the survey object.

A total of 400 questionnaires were sent out by mail to gas stations in the provinces of Jiangsu, Hunan, Guangdong, and Hebei. These provinces represent eastern China, middle-western China, southern China, and middle China, respectively. Since the corporation is an oligarch in China, thus in all these provinces, the China Petroleum & Chemical Corporation is the main gas supplier and the gas stations of China Petroleum & Chemical Corporation are also popular. According to this situation, we can claim that the data from these provinces can present the situation of China Petroleum & Chemical Corporation nationwide. In the survey, 259 of them were returned, with a return rate of 64.75%. Since the relationship between self-operated gas stations and Sinopec falls into the category of a company’s internal relationship, self-operated gas stations are excluded from the samples used in this study. After removing some incomplete or obviously incorrect questionnaires (e.g., giving 10 in the 5-point-scale or giving multiple scours in a same question item), 105 proper questionnaires are acquired for this study. Among them, jointly operated gas stations account for 46.2%, chartered gas stations 17.9%, and social gas stations 34.9%. Among the valid samples, gas stations with an annual sales volume of petroleum products below 2,500 tons account for 17.3%; those with a volume of 2,501–5,000 tons account for 38.7%; those with a volume of 5,001–7,500 tons account for 22.7%; those with a volume of 7,501–10,000 tons account for 10.7%; and those with a volume over 10,000 tons account for 10.6%.

### Variable Measurement

The measurement scales of each research variable in the questionnaire are from existing literature, and some of them are adjusted according to the specific situation of Sinopec and gas stations. Before designing the questionnaire, we conducted face-to-face in-depth interviews with dealers of non-self-operated gas stations to ensure the validity of the questionnaire. The five-point Likert scale is used to mark the measurements, where 1 stands for “totally disagree” and 5 stands for “totally agree.” The main measurement variables include dealer’s dependence, supplier’s dependence, the use of coercive power, the use of non-coercive power, and channel satisfaction. Table 1 shows the measurements of the main variables, reliability test results, and the source of the scale. Among them, the measurements of dealer’s dependence are based on the research by Heide (1994) and Lusch and Brown (1996), and supplier’s dependence is based on the research by Heide (1994) and Kumar et al. (1995). The measurements of the use of coercive power and use of non-coercive power are based on the research by Kale (1986) and Kumar et al. (1998). Measurements of channel satisfaction are based on the research by Cannon and Perreault (1999).

**TABLE 1:** Research variables and measurements.

Research variables and measurements	Standardized load	Cronbach’s alpha	Source of scale
**Dealer’s dependence**			
1. We can only buy oil from Sinopec.	0.773	0.859	Heide, 1994; Lusch and Brown 1996
2. If we discontinued our business relationship with Sinopec, we would find it difficult to find alternative suppliers.	0.757		
3. It’s a huge cost to lose Sinopec.	0.804		
4. It is difficult for us to switch to another supplier.	0.722		
5. Sinopec petroleum products account for a high percentage of all sales at our gas stations	0.730		
**Supplier’s dependence**			
1. Sinopec relies on us because of our position in the local retail market	0.730	0.887	Heide, 1994; Kumar et al., 1995
2. If Sinopec discontinued our business relationship, it would find it difficult to find alternative local gas stations of a similar scale.	0.792		
3. In the local market, Sinopec would suffer heavy losses if it lost our gas station.	0.830		
4. It is very difficult for Sinopec to find another gas station equivalent to ours.	0.739		
5. If we discontinued cooperating with Sinopec, Sinopec’s local sales would drop significantly	0.865		
**Use of coercive power**			
1. Sinopec often implies that if their requirements or regulations were not complied with, they would stop supplying or even cancel the dealership.	0.788	0.934	Kale, 1986; Kumar et al., 1998
2. Sinopec often reminds us that if we did not comply with their requirements or regulations, we would not receive their preferential policies (such as rewards, etc.)	0.836		
3. Sinopec often implies that if their requirements or regulations were not complied with, they would raise the price of their petroleum products.	0.799		
4. Sinopec often implies that if their requirements or regulations were not complied with, they would reduce the supply of their petroleum products.	0.977		
5. Sinopec often threatens to resort to legal means if their requirements or regulations were not complied with.	0.893		
**Use of non-coercive power**			
1. Sinopec can give us effective management advice, and we are willing to do it as recommended by Sinopec.	0.819	0.917	Kale, 1986; Kumar et al., 1998
2. Sinopec is a well-known brand in the market and we are willing to distribute products with Sinopec’s brand.	0.884		
3. Our business philosophy is very similar to Sinopec’s, so we are willing to do what Sinopec expects.	0.861		
4. We admire Sinopec’s way of doing business and are willing to be guided by it.	0.870		
**Channel satisfaction**			
1. Overall, we feel satisfied with Sinopec.	0.877	0.928	Cannon and Perreault (1999)
2. We appreciate Sinopec’s contribution to our company.	0.824		
3. We are very pleased to cooperate with Sinopec.	0.889		
4. We do not regret our decision to cooperate with Sinopec.	0.908		
5. If we were to choose again, we would still choose Sinopec.	0.797		

Since we could not find existing literature about how to measure the symmetry directly, this study measures dependence symmetry mainly by comparing the dealer’s dependence with the supplier’s dependence. Firstly, we calculate the average scour of the items in each part, and then we calculate the difference between the two parts and get the absolute value. According to this measurement, it can be claimed that the smaller the absolute value, the greater the dependence symmetry between the dealer and the supplier. Finally, since we use the 5-point-scale, the symmetry measuring result for each individual is obtained by subtracting 5 from the absolute value.

### Validity Testing

Convergent validity is measured using the recommendations from Fornell and Larcker (1981) to compare whether the extracted variances of all individual indicators and constructs are greater than the measurement error. At this time, the extracted variances of individual measurement indicators are larger than the measurement error; second, if the average variance extracted (AVE) of the construct is greater than 0.5, then the explained variance of the construct is more than 50%, i.e., the extracted variance of the construct is greater than the measurement error. It can be seen from Table 2 that the AVE values of the five research variables in this study are between 0.574 and 0.742, which is greater than the minimum requirement of 0.5, thus meeting the second requirement. In summary, the measurement model of this study can be considered to be with convergent validity.

**TABLE 2:** Mean, standard deviation, correlation coefficient, and AVE values of relevant variables in this study.

	Mean	Standard deviation	1	2	3	4	5
1. Dealer’s dependence	3.626	0.945	(0.574)				
2. Supplier’s dependence	3.285	0.985	0.507	(0.629)			
3. Use of coercive power	2.734	1.099	−0.456	0.031	(0.742)		
4. Use of non-coercive power	3.772	0.923	0.728	0.335	−0.289	(0.738)	
5. Channel satisfaction	4.03	0.827	0.7	0.141	−0.342	0.631	(0.740)

The most widely used method of discriminant validity testing is to examine whether the AVE values of all factors are greater than the square of the correlation coefficient (Fornell and Larcker, 1981; Shook et al., 2004). From Table 2, the AVE values of the five research variables in this study are between 0.574 and 0.742 with a minimum of 0.574; and the correlation coefficient among the factors is between −0.456 and 0.728 with a maximum is 0.728. The maximum square of the correlation coefficient among the variables is 0.530 (0.728 × 0.728), which is smaller than 0.574, the minimum AVE value. Therefore, the AVE values of all variables are greater than the square of the correlation coefficient among the variables, thus the measurement model of this study is considered to be of relatively high discriminant validity.

## Hypothesis Testing, Analysis, and Discussion

### Testing Method

This study adopts multiple linear regression as its method of data analysis. The model established is as follows:


(1) $$P_{c}=\beta_{1}\,D_{b}+z_{1}+e_{1}$$

(2) $$P_{c}=\beta_{2}\,D_{b}+\beta_{3}\,S+z_{2}+e_{2}$$

(3) $$P_{\mathrm{nc}}=\beta_{4}\,D_{b}+z_{3}+e_{3}$$

(4) $$P_{\mathrm{nc}}=\beta_{5}\,D_{b}+\beta_{6}\,S+z_{4}+e_{4}$$

(5) $$S\,A=\beta_{7}\,P_{c}+\beta_{8}\,P_{\mathrm{nc}}+z_{5}+e_{5}$$

where *P*_*c*_ stands for the use of coercive power; *P*_*nc*_ stands for the use of non-coercive power; *D*_*b*_ stands for a dealer’s dependence; *S* stands for dependence symmetry; *SA* stands for channel satisfaction. In the above equations *z*_*1*_, *z*_*2*_, *z*_*3*_, *z*_*4*_, and *z*_*5*_ are constants, and *e*_*1*_, *e*_*2*_, *e*_*3*_, *e*_*4*_, and *e*_*5*_ represent for the error term of the models. To avoid multicollinearity, this study refers to the method adopted by Jaccard and Wan (1995), in which all variables are centralized by means of deviation from average. The results of multicollinearity show that all VIF values are far below 10, indicating that the multicollinearity issue is unlikely to affect the analysis.

## Results and Discussion

To test H1, regression analysis on the data was performed with the use of coercive power (*P*_*c*_) as the dependent variable and dealer’s dependence (*D*_*b*_) as the independent variable. The results are shown as Model 1 in Table 3. It can be seen from the table that the dealer’s dependence holds a notable negative effect on the supplier’s use of coercive power (β_1_ = −0.444, *p* < 0.001), and this effect is still notable after taking into consideration the cross-terms of dependence symmetry and dealer’s dependence (Model 2) (β_2_ = −0.420, *p* < 0.001). Therefore, the empirical test results support H1. Similarly, to test H2, regression analysis on the data was performed with the use of non-coercive power (*P*_*nc*_) as the dependent variable and dealer’s dependence (*D*_*b*_) as the independent variable. The results are shown as Model 3 in Table 3. It can be seen that the dealer’s dependence is positively correlated with the supplier’s use of non-coercive power (β_4_ = 0.581, *p* < 0.001), and this effect is still notable after taking into consideration the cross-terms of dependence symmetry and dealer’s dependence (Model 4) (β_5_ = 0.576, *p* < 0.001). Hence, H2 is supported by the regression results above. The fact that H1 and H2 are supported indicates that as the dealer becomes more reliant on the supplier, the supplier will correspondingly use less coercive influence strategies on the dealer, such as threats, defenses, and legal means, and it will use more non-coercive influence strategies, such as requests, information exchange, and suggestions. Especially in the context of China, where dependence is not contemptible, the dealer would actively look for someone to rely on (backers), and the supplier would regard the dealer’s dependence as part of their own strength (Zhuang and Zhou, 2004). When the dealer becomes more dependent, the supplier would regard the dealer as one of its own business systems, and hence gradually abandon the unfriendly use of coercive power and use more non-coercive power to influence, help, and support the dealer.

**TABLE 3:** Statistical results of multiple regression.

Variable	Model				
	
	(1)	(2)	(3)	(4)	(5)
	
	Use of coercive power	Use of coercive power	Use of non-coercive power	Use of non-coercive power	Channel satisfaction
Dealer’s dependence	−0.444***	−0.420***	0.581***	0.576***	
Dealer’s dependence * dependence symmetry		0.292*		–0.047	
Use of coercive power					−0.119*
Use of non-coercive power					0.513***
*R*^2^	0.155	0.198	0.522	0.524	0.368
Δ*R*^2^		0.043*		0.002	
F	18.922***	12.631***	112.425***	56.172***	29.655***

*p < 0.05, ***p < 0.001.

To test H3, regression analysis on the data was performed with the use of coercive power (*P*_*c*_) as the dependent variable and dealer’s dependence (*D*_*b*_), along with the cross-terms of the dealer’s dependence (*D*_*b*_) and dependence symmetry (*S*), as the independent variables. The results are shown as Model 2 in Table 3. The cross-terms in Model 2 are positive and reach a notable level (β_3_ = 0.292, *p* < 0.05), and dependence symmetry plays a significant role in moderation. The hierarchical regression results from Model 1 to Model 2 further suggest the moderating role dependence symmetry plays in dealer’s dependence and supplier’s use of coercive power (Δ*R*^2^ = 0.43, *p* < 0.05). The positive cross-term coefficient indicates that as dependence symmetry between the supplier and the dealer increases, the influence of the dealer’s dependence on the supplier’s use of coercive power diminishes, hence H3 is supported. This indicates that when the dependency symmetry is low, as dealer’s dependence increases, the supplier will have more resources that are valuable for the dealer, and the dealer will be even less likely to deviate from the supplier’s demands and wishes. Therefore, the supplier will feel less necessary to use coercive influence strategies. When dependence symmetry is high, the dealer would depend less on the supplier because of the advantage in specific respects. It also has resources that are of great value to the supplier, and it may deviate from the cooperation with the supplier by taking advantage of these resources. Therefore, the supplier has to use more coercive power like commitments, legal means, and defenses to stabilize and regulate their cooperation.

To test H4, regression analysis of the data was performed with the use of non-coercive power (*P*_*nc*_) as the dependent variable and dealer’s dependence (*D*_*b*_) along with the cross-terms of the dealer’s dependence (*D*_*b*_) and dependence symmetry (*S*) as the independent variables. The results are shown as Model 4 in Table 3. The negative cross-term coefficient of dealer’s dependence and dependence symmetry in Model 4 indicates that as dependence symmetry increases, the influence of the dealer’s dependence on the supplier’s use of non-coercive power diminishes. However, the influence does not reach a notable level (β_6_ = −0.47, *p* = 0.489). The hierarchical regression results from Model 3 and Model 4 also suggest the moderating role dependence symmetry plays in dealer’s dependence and supplier’s use of non-coercive power is not notable (Δ*R*^2^ = 0.002, *p* < 0.498); hence H4 is not supported. Further explanations are as follows: first, as opposed to the use of coercive power, the use of non-coercive power is a preferred influential strategy for the source company to try to influence the target company because it conduces more to maintaining a harmonious and stable channel relationship network. Therefore, regardless of the intensity of dependence symmetry, the more dependent the target company is, the stronger the source company feels it is relied upon, which gives rise to a closer relationship and more willingness to use non-coercive power. Second, in the case of high dependence symmetry, the source company has very limited non-coercive power, but the source company can keep the intensity of the use of non-coercive power by more frequent use of power, such as frequent information exchange and the instillation of product sales strategies, etc. Both reasons may lead to the empirical conclusion of weak and inconspicuous moderation in this study.

To test H5 and H6, regression analysis was performed with channel satisfaction (*SA*) as the dependent variable, and the use of coercive power (*P*_*c*_) and the use of non-coercive power (*P*_*nc*_) as the independent variables. The results are shown in Model 5 in Table 3. It can be seen that the supplier’s use of coercive power diminishes channel satisfaction (β_7_ = −0.119, *p* < 0.05), while its use of non-coercive power improves channel satisfaction (β_8_ = 0.513, *p* < 0.05). Hence H5 and H6 are confirmed. Consistent with the findings of previous studies that the use of non-coercive power improves channel satisfaction, this study also shows quite a strong correlation between the two; however, existing studies drew inconsistent conclusions on the relationship between the use of coercive channel power and channel satisfaction. In the context of oriental cultures, in particular, some empirical research found that the use of coercive channel power would significantly influence the channel satisfaction in a positive way. Zhang and Marsha (2004) explained the positive relationship with the vertical social relations in oriental commercial systems, which feature on command and obedience. Therefore, Orientals are more adaptable and accustomed to the use of coercive power in commercial relations. Although this study does not support this conclusion, the negative relation between the supplier’s use of coercive power and the dealer’s channel satisfaction in this study is significantly weaker than the influence of the use of non-coercive power on channel satisfaction. More importantly, the intensity of this relationship is also much weaker than conclusions drawn in the context of western cultures. For instance, Frazier and Summers’ (1986) study concluded that the influence between dealer’s channel satisfaction and threats, legal defenses, and commitments used by manufacturers were −0.32, −0.29, and −0.25, respectively. In the study of Gaski and Nevin (1985), the correlation coefficient of the use of coercive power and channel satisfaction was −0.305. Therefore, the conclusion of this study shows that, in the context of China, the social relations focusing on loyalty, command, and obedience do make Orientals feel different from westerners about coercive power.

## Conclusion

This study draws on the SCP research paradigm in the theory of industrial organization and believes that the basic source of supplier’s use of channel power is the dealer’s dependence structure, with the result being the quality of the channel relationship. Starting from the concept of channel dependence, this study divides channel dependence structure into two aspects: channel dependence and dependence symmetry. It divides the use of channel power into the use of coercive channel power and the use of non-coercive channel power. It studies channel dependence structure’s impact on the use of channel power, and ultimately its effect on channel satisfaction. The findings of this study hold significant values, both theoretically and practically.

The theoretical value of this study is mainly presented in two aspects: (1) there is a great inconsistency in existing studies on channel dependence and the use of power in terms of the relationship between channel dependence and the use of channel power. This study argues that the reason for this is the inconsistent definition and measurement of channel dependence in existing studies. In particular, existing studies often regarded channel dependence simply as A’s dependence on B, rather than considering B’s dependence on A at the same time. In other words, channel dependence was not considered from a structural perspective. This study introduces the concept of dependence symmetry on the basis of channel dependence and believes that channel dependence and dependence symmetry constitute a channel dependence structure. The research findings show that channel dependence symmetry plays an important role in moderating the relationship between channel dependence and the use of non-coercive power. As channel symmetry increases, the dealer’s channel dependence will have less effect on the supplier’s use of non-coercive power. (2) Inconsistencies exist in the conclusions of existing studies on the relationship between the use of channel power and channel satisfaction. In particular, some studies in the context of oriental cultures found a positive correlation between the use of coercive power and channel satisfaction. Although the findings of this study do not support this viewpoint, it is found that the relationship between the use of coercive power and channel satisfaction is much weaker than that in the context of western cultures. This finding shows that the relationship between the channel structure and channel members is closely correlated to specific cultural and social backgrounds (Olsen and Granzin, 1990).

The practical value of this research is presented in the following three aspects: (1) good quality of channel relationship is a common goal pursued by dealers and suppliers. For suppliers, in particular, dealers are an important link to pass on the value of their products and services. This study found that suppliers’ use of coercive power is negatively correlated with the dealer’s channel satisfaction, while the use of non-coercive power is positively correlated with channel satisfaction. Therefore, when trying to influence dealers’ behavior, suppliers should mainly use non-coercive power and be careful in using coercive power. Suppliers can, for instance, put forward effective ways of doing business for dealers or instill business philosophy into dealers and get recognition from them, etc. Specifically, in the petrochemical industry, to obtain a satisfactory relationship with the on-self-operated gas stations (dealers), the company like China Petroleum & Chemical Corporation should try to make the dealers agree with corporate values or even share the same goal instead of forcing them. (2) This study finds that the use of non-coercive channel power is based on heavy dependence of dealers. From this perspective, suppliers need to constantly increase the dealer’s dependence on themselves if they are to improve the quality of the channel relationship. From the meaning of dependence itself, it is required that suppliers should secure more resources and capabilities that are important to dealers, such as providing differentiated products, building strong brands, building expert power, increasing the cost of switching supplier for dealers, etc. For the companies in the petrochemical industry, although the industry in China is oligarch, suppliers like China Petroleum & Chemical Corporation should still try to enhance themselves to equip with competitive capabilities and resources to provide basic conditions to use non-coercive channel power to increase channel satisfaction. (3) When dealers are highly dependent, if the dependency asymmetry is great, suppliers will increase the use of coercive power, which will in turn diminish channel satisfaction. Therefore, in addition to adopting the strategy of making dealers more dependent, suppliers must make themselves less dependent on dealers by strengthening their own distribution channel system. For example, petrochemical companies can increase the number of their own gas station outlets to be less dependent on the dealers so that the dependency asymmetry might be under control and the channel satisfaction might be increased. Also, companies in other industries like the telecom industry can diminish their dependence on social distribution channels by increasing the number of their own service centers. Suppliers can also diminish their dependence by actively expanding the existing distribution channel mode, such as telecom companies further diminishing their dependence on social distribution channels by expanding the construction of electronic channels.

The limitations and future research directions of this study are as follows: (1) This study takes dealers (non-self-operated gas stations) as the research object to study the relationship between supplier’s and dealer’s dependence structure, the use of channel power, and channel satisfaction. Due to the limitations of the research objects, it is difficult to avoid deviation from the common method. Besides, this one single side data shows a limitation in discussing two side’s channel relations since the perception of the use of power and satisfaction by two sides in the channel could be influential. Therefore, in the future, surveys can be done on both suppliers and dealers to obtain dyadic data, so as to reflect the status of channel relationship in a true and comprehensive fashion. (2) The petroleum product sales industry involved in this study holds typical features of a monopoly. The channel relationship in different market structures may differ considerably. In future, studies can be done on the channel relationship in other industries and other types of market structures to explore market structure’s influence as a variable. (3) This article studies the moderating role channel dependence symmetry plays between channel dependence and the use of power in the context of China. Further studies are needed to assess whether relevant conclusions can be applied in the context of western cultures.

## Data Availability Statement

The datasets presented in this article are not readily available because data could not be shared due to promise of confidentiality and no-sharing with the respondents. Requests to access the datasets should be directed to XD, dengxy@btbu.edu.cn.

## Author Contributions

JT: conceptualization, methodology, and writing – original draft preparation. XD: validation, investigation, data analysis, and financial sponsor. QS: data collection, writing – review, editing, and validation. All authors contributed to the article and approved the submitted version.

## Conflict of Interest

The authors declare that the research was conducted in the absence of any commercial or financial relationships that could be construed as a potential conflict of interest.

## Publisher’s Note

All claims expressed in this article are solely those of the authors and do not necessarily represent those of their affiliated organizations, or those of the publisher, the editors and the reviewers. Any product that may be evaluated in this article, or claim that may be made by its manufacturer, is not guaranteed or endorsed by the publisher.
